# Salmonella Subcutaneous Abscess: A Rare Complication Post Salmonella Gastroenteritis

**DOI:** 10.7759/cureus.52945

**Published:** 2024-01-25

**Authors:** Dominic Nguyen, Zhuang Hui Mark Le, Saranga Ranasinghe

**Affiliations:** 1 General Surgery, Redland Hospital, Redland, AUS

**Keywords:** salmonella complications, chest wall swelling, atypical infection, chest wall abscess, salmonella infection

## Abstract

*Salmonella* is a gram-negative bacilli bacterium that can infect and colonize humans, causing a wide range of clinical manifestations. The most common manifestation is gastroenteritis, usually after ingestion of undercooked and ill-prepared food, particularly in developing countries. Indonesia is among those reported to have a high incidence of *Salmonella* infection. Uncommonly, extraintestinal manifestations can affect distant body sites, either during or after the episode of bacteremia. This case report presents a rare case of a healthy 19-year-old female who developed an atypical chest wall abscess colonized by *Salmonella* in the context of experiencing gastroenteritis three weeks prior on her return from Bali, Indonesia. This case highlights the indolent course associated with a *Salmonella* chest wall abscess with a discussion of the current literature and management.

## Introduction

*Salmonella *is a gram-negative bacilli bacterium and is a worldwide health concern with the most common manifestation being gastroenteritis. The main source of this infection is food, with the highest incidence within developing countries due to poor hygiene and lack of clean water [1]. Patients generally suffer from diarrhea, fever, and abdominal pain, and the illness is generally self-limiting with no specific intervention. Bacteriaemia is a serious and potentially fatal complication from nontyphoidal *Salmonella *infection that can occur in up to 5% of patients, with the incidence being higher in immunocompromised patients [2]. Several extra-intestinal manifestations include (but are not limited to) the involvement of the cranial nervous system, cardiovascular and pulmonary systems, musculoskeletal system, hepatobiliary, and genitourinary systems [3,4]. This case report presents an atypical case of *Salmonella *chest wall subcutaneous abscess in a 19-year-old female with a history of gastroenteritis in the preceding three weeks with a discussion of the current literature and management.

## Case presentation

A 19-year-old female presented to the emergency department with a five-day history of right lower chest wall pain overlying her sixth costal cartilage. She had no features of systemic illness and denied any trauma or insect bites. She had no past medical or surgical history and denied any smoking, alcohol use, or recreational substance. Initially thought unrelated, this was after recently suffering from an episode of gastroenteritis upon return from a trip to Bali, Indonesia. The episode of gastroenteritis was self-limiting and required no hospital admission, however, she did lose 5-10kg. On examination, she was focally tender in the right lower chest wall over the lower rib. There was no associated swelling or skin changes. The patient’s inflammatory markers and radiological investigations (including a chest x-ray) were unremarkable. A diagnosis of costochondritis was made, and she was subsequently discharged on non-steroidal anti-inflammatories.

**Figure 1:**
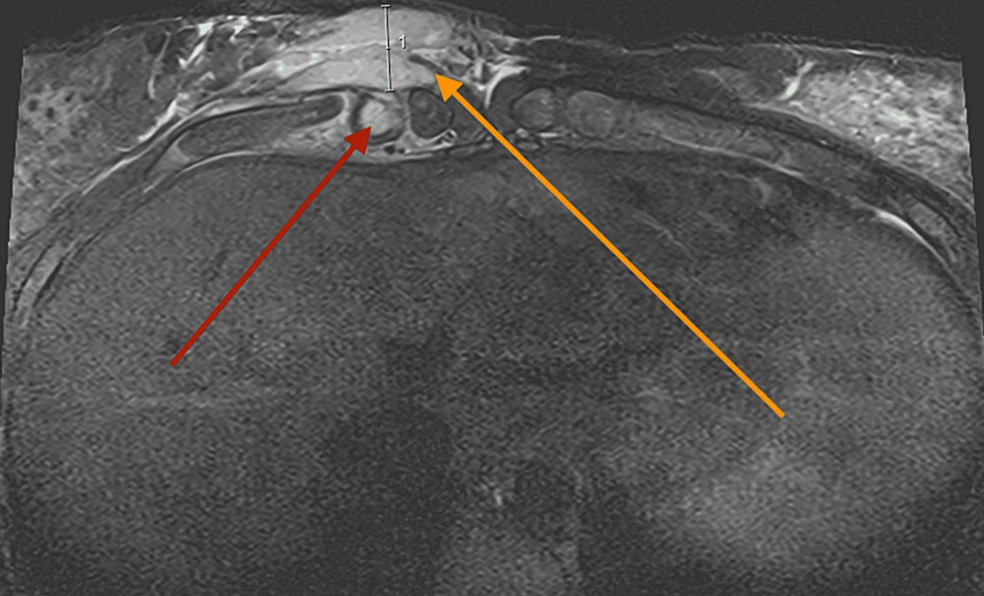
MRI chest wall (axial). Infective chondritis with associated subcutaneous inflammation. Red arrow: enhancing T2 hyperintense focus measuring 13x5mm, involving the mid costal cartilage on the right Orange arrow: associated subcutaneous inflammatory phlegmon/abscess (length: 19.61mm)

**Figure 2:**
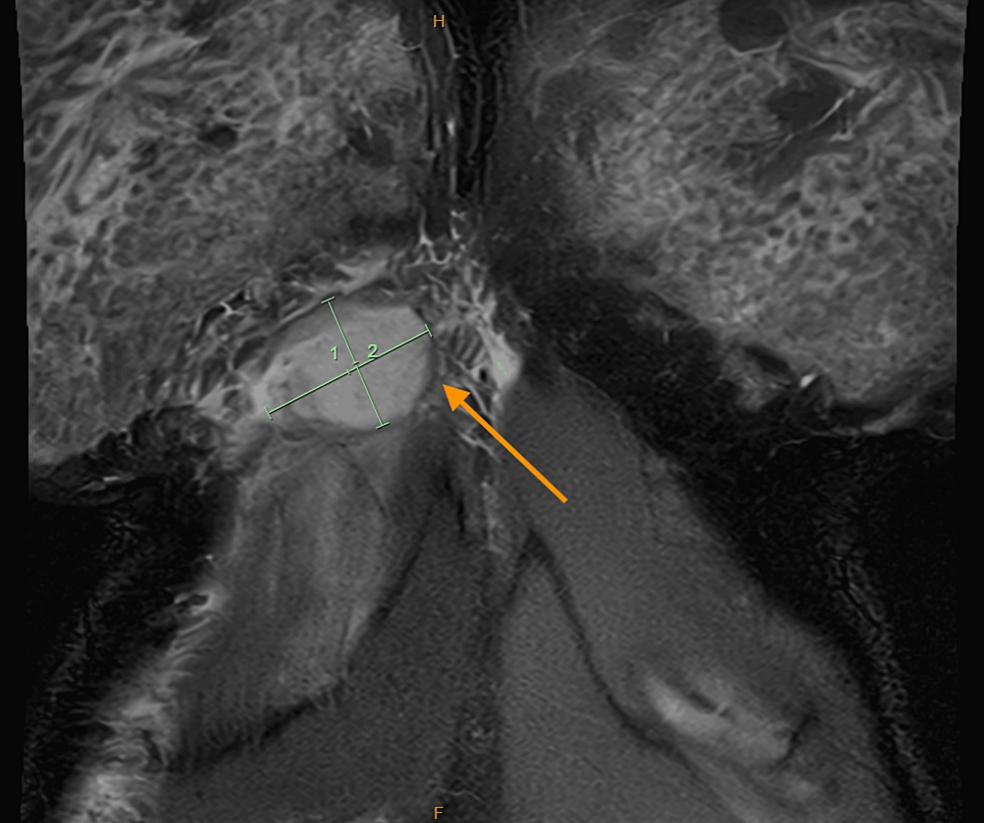
MRI chest wall (coronal). Inflammatory phlegmon/abscess at right chest wall overlying 6th costal cartilage. Length: 40.18mm (1) x 30.42mm (2)

**Figure 3:**
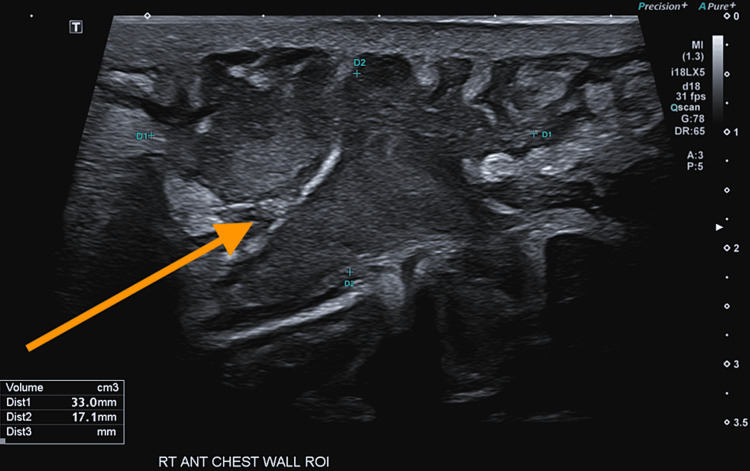
USS chest wall. Collection extending deep toward the costal cartilage with associated soft tissue cellulitis. Collection measuring 7x33x17mm (23mL).

Two weeks later, the patient developed a recurrence of the pain with new associated swelling. She initially represented her general practitioner and was prescribed a course of Augmentin Duo Forte. Due to persistent symptoms despite oral antibiotic treatment, the patient was presented to the emergency department and a surgical review was sought. Again, the patient had no features of systemic illness. On examination, a 4.0cm x 3.0cm firm, erythematous, and indurated mass was appreciated on her right chest wall, overlying her sixth costal cartilage. There was no point of maximal fluctuant or associated lymphadenopathy. Hematological investigations demonstrated normal inflammatory markers. Various forms of imaging were performed to delineate the nature and characteristics of the chest wall lesion. A computed tomography (CT) of the chest showed a right-sided costal chondral infection, with no obvious drainable collection. Magnetic resonance imaging (MRI) of the chest also demonstrates features of infective chondritis involving the mid-costal cartilage. There was an associated subcutaneous inflammatory phlegm or abscess (Figure 1 axial, Figure 2 coronal). The subsequent US of the right chest wall demonstrated the development of a 33mm x 17mm x 7mm (23mL) drainable collection with mobile internal echoes, extending toward the costal cartilage with associated soft tissue cellulitis (Figure 3).

The patient subsequently underwent surgical debridement which showed a single large cavity of necrotic debris but no purulent discharge. The cavity did not extend to the underlying rib, and the intercostal fascia was intact. Cultures of the wound demonstrated *Salmonella* spp. non-typhoid. She was consequently discharged on azithromycin, with further follow-up in the surgical outpatient department four weeks later and demonstrated complete resolution of the disease.

## Discussion

Subcutaneous abscesses are a common presentation that requires prompt surgical debridement. The pathophysiology is widely understood to be secondary to bacterial overgrowth in the dermis and subcutaneous tissues leading to a localized cavity filled with purulent material. This is usually precipitated by a breach in the skin or via hair follicles. The most common pathogens are *Staphylococcus aureus* and *Streptococci *spp. on the trunk or limbs and a combination of aerobes and anaerobes in the perineal region [5].

This case presents an unusual cause for subcutaneous abscesses, with the etiology likely secondary to *Salmonella* spp., disseminated via a suspected bacteremia source. The patient denied any skin breach overlying the abscess, with the only other symptoms preceding an episode of gastroenteritis in the month prior. The patient described symptoms in keeping with bacteremia; however, given the self-limiting nature, no investigations were conducted at the time to confirm this diagnosis. With the high incidence rate of *Salmonella* in Bali, we speculate that the patient developed a gastroenteritis infection, and acknowledge that no investigation was done at the time to confirm this diagnosis [1]. With the subsequent cultures grown from her abscess drainage, we assume that the bacteria had a hematogenous spread.

Upon review of the literature, very few cases of subcutaneous abscess with *Salmonella* have been reported. A case was reported in 2022 in which a 39-year-old healthy male developed an anterior chest wall abscess after an episode of *Salmonella* gastroenteritis and bacteremia which required intravenous antibiotic therapy [6]. Similar to our case, he developed a chest wall abscess several months after his episode of gastroenteritis, which also required surgical debridement. To our knowledge, this is the only other documented subcutaneous manifestation as a sequela of *Salmonella*. This represents a second rare case of the novel sequelae of *Salmonella* infection. A high index of suspicion is required to further evaluate and manage these unusual cases of atypical subcutaneous abscesses.

## Conclusions

Subcutaneous abscess is a common presentation that affects many different people. This case was atypical in that the abscess was secondary to a *Salmonella *infection, likely disseminated from a hematogenous route after an episode of gastroenteritis, with no history of skin breach prior to the development of the abscess. The patient's symptoms were also unusual in that the abscess was slow to progress, with a protracted history of having chest wall discomfort but no obvious abnormality on examination. This case highlights that a thorough history should be taken with a high index of suspicion of where the infection is coming from to exclude any other sources. Once a diagnosis is made, prompt surgical debridement should be completed and to determine the underlying organisms, samples should be taken including mycobacterium, atypical and fungal cultures.
